# Potential Drug–Drug Interactions and Related Factors among Geriatric Outpatients of a Tertiary Care Hospital

**DOI:** 10.3390/geriatrics8060111

**Published:** 2023-11-14

**Authors:** Tippayavadee Wannawichate, Panita Limpawattana

**Affiliations:** 1 Department of Pharmacy Service, Srinagarind Hospital, Faculty of Medicine, Khon Kaen University, Khon Kaen 40002, Thailand; tippra@kku.ac.th; 2 Division of Geriatric Medicine, Department of Internal Medicine, Faculty of Medicine, Khon Kaen University, Khon Kaen 40002, Thailand

**Keywords:** drug–drug interactions, elderly, outpatient, prevalence

## Abstract

(1) Background: Drug–drug interactions (DDIs) possess the potential to lead to a range of clinically significant consequences in the older population. (2) Aims: To investigate the prevalence and associated factors of DDIs among older patients within an outpatient setting of a university hospital. (3) Methods: This is a descriptive analysis of patients aged ≥65 years, who received a minimum of two medications. The electronic medical records were obtained from the outpatient clinic of a tertiary care hospital between November 2021 and November 2022. The outcomes were analyzed using descriptive and regression analysis. (4) Results: The study enrolled 10,877 patients, with a mean age of 74.3 ± 6.8 years. The prevalence of major DDI was 36.8%. Factors associated with major DDI were age (odds ratio [OR] 1.03), female sex (OR 1.23), polypharmacy (OR 2.27–13.78), metabolic disease (OR 1.89), psychiatric disorder (OR 1.79), cardiovascular disease (OR 1.51), musculoskeletal disease (OR 1.37), central nervous system disease (OR 1.24), and tuberculosis (OR 0.18). There was a significant difference observed in the primary healthcare facilities for emergency medicine (OR 1.72), orthopedics (OR 1.36), internal medicine (OR 1.29), and radiology (OR 0.45). (5) Conclusions: Major DDI was prevalent among older patients receiving care at outpatient settings. Several factors linked to major DDIs were identified. Developing appropriate strategies to improve the prescription process and avoid any missed interactions with geriatric patients is recommended.

## 1. Introduction

Drug–drug interactions (DDIs) refer to the pharmacologic or clinical response that arises from the administration of a drug combination, which deviates from the expected effects of the individual agents [1]. Such interactions can lead to a range of clinically significant outcomes, including increased outpatient visits, prolonged therapy, hospitalization, elevated healthcare cost, and heightened mortality risk. Particularly, older patients are at a greater risk of hospital admissions due to DDIs, with rates ranging from 4.8% to 19.6% [2,3]. DDIs are highly prevalent in older patients. The prevalence of DDIs varies widely, ranging from 5.3% to 100%, depending on different settings and the tools used for assessment [2,4,5]. For example, a cross-sectional study conducted among geriatric outpatients in a tertiary care hospital in China revealed that the prevalence of DDIs was 32.22%, 32.93%, and 22.62% based on the Lexicomp^®^, Micromedex^®^, and DDInter, respectively [4]. In an outpatient setting under the local health authority of Leco, Italy, an observational study was conducted on individuals aged 65 or older to determine the prevalence of potential DDIs using the Italian interaction database and revealed a prevalence rate of 16% for these interactions [6]. Another study conducted in Belgium from the OPERAM trial, reported the point-prevalence rates of DDIs using an international consensus list of potentially clinically significant DDIs in older people at discharge, 2, 6, and 12 months of 58%, 57%, 56%, and 57%, respectively. Drugs that affect potassium concentrations, centrally acting drugs, and antithrombotic drugs were involved in the top five pharmacodynamic DDIs, which accounted for 80% of all DDIs before and after hospital admission [7]. A retrospective observational study was performed in a community setting of Qatar, which found that the prevalence of at least one potential DDI was 31.9% using the Lexicomp^®^ database, and 17.9% using the Micromedex^®^ database [8]. A systematic review and meta-analysis conducted among community-dwelling older adults showed a pooled DDI prevalence of 16.6%, with cardiovascular drugs (ACE inhibitors-potassium sparing diuretic; amiodarone-digoxin; and amiodarone-warfarin) being the most commonly paired drugs [5].

Advanced age and polypharmacy have been recognized as significant risk factors for the occurrence of DDIs. Furthermore, patients with chronic diseases such as diabetes, chronic obstructive pulmonary disease, stroke, osteoporosis, and depression are at an increased risk of DDI occurrence due to multiple comorbidities, advanced age, polypharmacy, chronic therapeutic regimens, frequent outpatient visits, frequent hospital admissions, frequent modification of therapy, prolonged treatment, and multiple prescribers [3,4,6]. The prevalence of DDIs is associated with the visited department, with internal medicine (9.2%) and cardiology (2.6%) having higher rates compared to other outpatient department specialties [9]. However, one study identified neurology visits as a significant risk factor for DDIs [4].

The examination of DDIs and its associated factors in Thailand is restricted, particularly in tertiary care facilities where the complexity of patients is elevated. Prompt recognition of DDIs can prevent the omission of such interactions and promote the use of geriatric-friendly medication. Thus, this study endeavors to assess the prevalence and factors linked to DDIs among older patients in outpatient settings of a tertiary care hospital.

## 2. Materials and Methods

### 2.1. Study Design and Setting

A descriptive, retrospective analysis was carried out at Srinagarind Hospital, Khon Kaen—a tertiary care facility and a University Hospital situated in the Northeastern region of Thailand. It is a medical hub that operated under the Faculty of Medicine of Khon Kaen University. In the year 2022, the cumulative count of individuals seeking outpatient care reached 1,084,048 cases, with 289,430 cases (equivalent to 26.7%) belonging to patients aged 65 years or older [10]. During the period from November 2021 to November 2022, all prescriptions for the patients were collected consecutively from the outpatient clinic. Information about the patients was retrieved from the hospital information system. During the collection process, standard care according to the National regulations was taken to ensure patient anonymity and data confidentiality. 

### 2.2. Populations

Patients aged 65 years or above, who were administered a minimum of two medications, were included in this study. The electronic medical record was obtained solely from the Special Medication Center. Topical medications, such as inhalers, creams, ointments, patches, and sprays, were excluded from this study.

### 2.3. Data Collection

The study obtained data using electronic medical records, which covered baseline characteristics and patient demographics including age, gender, number of prescribed medications, principal diagnosis, comorbidities, and primary medical service.

### 2.4. Assessment of DDIs 

The investigation of DDIs in prescriptions was carried out using the UpToDate^®^ (version 7.7.5, 19 May 2023), which is a database commonly utilized in clinical settings, is integrated into the UpToDate^®^ [11]. Within this database, there is an accompanying statement that qualifies the nature of the interaction(s) described in the monograph. Additionally, there may be an indication of the severity of the outcome and/or onset of an unmanaged interaction. The severity indicators encompass three levels: minor, moderate, and major. Minor effects are typically considered tolerable in most cases and do not require medical intervention. Moderate effects necessitate medical intervention to treat the effects but do not meet the criteria for the major. Major effects may result in death, hospitalization, permanent injury, or therapeutic failure [12].

### 2.5. Statistical Analysis

Descriptive statistics were used to illustrate the characteristics of the study sample. Continuous variables, including age and number of comorbid diseases, were expressed as mean values with standard deviation (sd), whereas categorical variables including gender, numbers of medication, principal diagnosis, and primary medical service were presented as the number of participants with a percentage. The factors that were associated with major DDI were assessed using univariate and multivariate logistic regression analysis. The findings were subsequently demonstrated by presenting the crude odds ratios (OR) and adjusted OR, along with a respective 95% confidence interval (CI). All statistical analyses were conducted using STATA version 10.0 (StataCorp, College Station, TX, USA).

## 3. Results

### 3.1. Prevalence of Drug–Drug Interactions

A total of 10,877 patients were enrolled in this study via electronic medical-record recruitment. The prevalence of major DDI and moderate DDI was found to be 36.8% (4004 patients) and 31.4% (3414 patients), respectively. The baseline characteristics of the studied population are shown in Table 1. The mean age of patients was 74.3 ± 6.8 years, with a predominance of males and approximately 44% exhibiting polypharmacy (defined as the use of five or more medications) [13]. Metabolic disease (diabetes, hypertension, and dyslipidemia) was the most common principal diagnoses. Patients with major DDI appeared to have a higher prevalence of polypharmacy. The three most associated drugs with major DDI were sulfonylurea vs. dipeptidyl peptidase-IV inhibitors (3.95%); followed by opioid analgesics vs. CNS depressants (2.86%); and salicylates vs. nonsteroidal anti-inflammatory agents (2.77%) (Table 2).

### 3.2. Predictor of Major Drug–Drug Interaction (DDI) among Older Patients at Outpatient Setting

Table 3 presents the factors that were linked to major DDI, as identified through univariate and multivariate regression analyses. Based on the results of the former, age, polypharmacy, presence of musculoskeletal disease, metabolic disease, cardiovascular disease, chronic kidney disease (CKD), central nervous system (CNS) disease, psychiatric disorder, tuberculosis, number of comorbidities, and the main medical service of internal medicine, surgery, palliative care, ENT clinic, radiology, and gynecology compared to general practitioner (GP) clinic were all found to be associated with major DDI. These factors were then entered in the multivariate analysis, which revealed that increasing age (OR 1.03), female sex (OR 1.13), polypharmacy (2.27–13.78), presence of musculoskeletal disease (OR 1.37), metabolic disease (OR 1.89), cardiovascular disease (OR 1.51), CNS disease (OR 1.24), psychiatric disorder (OR 1.79), tuberculosis (OR 0.18), and the main medical services of internal medicine (OR 1.29), orthopedics (OR 1.36), emergency medicine (OR 1.72), and radiology (OR 0.45) were all associated with major DDI (*p* < 0.05). It should be noted that the reference point for the main medical service was a GP clinic, given that its patients were less complex than those in other settings.

## 4. Discussion

Previous research studies examining DDIs amongst geriatric outpatients have exhibited highly varied outcomes. The heterogeneity of these findings may be attributed in part to divergences in the definition of DDIs (i.e., potential or clinically significant) and the criteria employed to evaluate them (e.g., varying databases, consensuses, and reference books) [4]. This research primarily concentrated on major DDI, considering the potential for severe clinical implications [12]. The current study revealed a major DDI prevalence of 36.8%, which is consistent with the findings of prior studies conducted in China with similar settings, specifically 32.22% and 30.29% [4,14]. The prevalence of clinically important drug–drug interactions at an outpatient clinic of a secondary care hospital in Thailand using the 2019 American Geriatric Society (AGS) Beers criteria was lower at 15.66%; however, the results could not be directly compared since the AGS Beers criteria only checks a low number of DDIs [15]. Sulfonylurea vs. dipeptidyl peptidase-IV inhibitors, opioid analgesics vs. CNS depressants, and salicylates vs. nonsteroidal anti-inflammatory agents were the top three common major DDIs in this study. These findings align with a similar study conducted in China, wherein drugs used in diabetes were deemed the most significant clinically, followed by psychotropics, anti-inflammatory, and anti-rheumatic drugs. However, the proportions noted in the Chinese study were much higher than those observed in our research (41.26% vs. 3.95%, 18.85% vs. 2.86%, and 10.38% vs. 2.77%, respectively) [4]. The reason behind this could be attributed to the fact that metabolic diseases such as diabetes and musculoskeletal ailments were the most commonly occurring chronic conditions in this study.

Various factors were found to be associated with an increased risk of major DDI in our research. These factors include advancing age, female sex, polypharmacy, underlying diseases of musculoskeletal disease, metabolic disease, cardiovascular disease, CNS disease, psychiatric disorder, and tuberculosis. Furthermore, the main medical services of internal medicine, orthopedics, and emergency medicine were also linked to an increased risk of major DDI as compared to that of a general practitioner clinic. However, radiology was identified as a protective factor. It is worth noting that advancing age was a significant factor, as expected. This can be attributed to the fact that older adults tend to be more susceptible to pharmacokinetic effects due to diminished hepatic and renal function [5,14,16]. However, one study reported the risk of major DDI was independent of advancing age. According to that study, patients aged 70–74 years exhibited a greater prevalence of DDIs as compared to those aged 80–89 years, which could be due to the cautious prescription practices adopted by medical practitioners for the latter age group [4]. In our research, it was found that women were at risk, which could potentially be attributed to the inherent nature of women. This is due to their heightened concern for their health and their tendency to seek healthcare services more frequently than men. Additionally, women tend to exhibit a greater recognition and experience of health issues, as well as an increased susceptibility to underlying gender-related psycho-social and behavioral factors, resulting in a higher perceived symptom burden. As a result, women tend to use a larger quantity of medications, which in turn leads to a higher number of potential DDIs when compared to men. However, the findings in previous studies have been inconsistent [14,17].

Polypharmacy was associated with clinical frailty in the earlier research [18], and it also remains a recognized risk factor for major DDIs in this finding, as previously reported. The risk is significantly greater when the number of medications exceeds 10, as compared to 5–9 medications (OR 13.78 vs. 2.27). However, it is important to note that polypharmacy does not automatically imply inappropriateness, as older patients often have multiple comorbidities that require necessary medications to achieve therapeutic responses. In clinical practice, prescribing as indicated, appropriate dose adjustment, and close monitoring are crucial. Furthermore, the incorporation of non-pharmacological strategies should be implemented for all patients to mitigate the adverse effects of DDIs [4,14,19].

The primary diagnosis of musculoskeletal disease, metabolic disease, cardiovascular disease, CNS disease, and psychiatric disorder increased the risk of major DDI, while tuberculosis was a protective factor. Due to the progressive deterioration in physiological function and metabolic processes that older patients experience, it is common for disorders related to musculoskeletal, metabolic, and neurodegenerative processes to be prevalent in this population [20,21]. These diseases are commonly treated with antidiabetic, anti-inflammatory, and psychotropic medications. Notably, the major DDI observed involve sulfonylurea versus dipeptidyl peptidase-IV inhibitors, opioid analgesics versus CNS depressants, and salicylates versus nonsteroidal anti-inflammatory agents. It is worth noting, however, that current screening methods for major DDI may be oversensitive, resulting in a high alert burden and frequent overrides of both clinically significant and insignificant alerts by clinicians [4]. The identification tool for detecting DDIs seems to constitute only a fraction of the overall process of ensuring high-quality prescribing practices. It is, therefore, imperative that the development of a more personalized approach to assessing the clinically significant risks of DDIs is undertaken, as this would significantly enhance the efficacy of efforts aimed at preventing potentially serious adverse reactions and improving the outcomes of pharmacotherapy [4]. Interestingly, antituberculosis chemotherapy decreased the risk of major DDI in this study. These findings are in contrast to those of the previous review, which highlighted the clinically significant interactions associated with drugs used for tuberculosis treatment [22]. One plausible rationale could be attributed to the efficacy of the Thai National Tuberculosis Control Program Guideline, which offers a contemporary and user-friendly manual aimed at mitigating negative consequences, discontinuation, and ultimately strengthening therapeutic success [23,24].

According to the multivariate analyses, patients from the emergency service exhibited the highest odds ratio for increasing the risk of major DDI, followed by the orthopedics and internal medicine services. The results support that drug-related adverse events are among the most frequently reported errors in the emergency department [25]. The emergency service is particularly susceptible to DDIs due to a host of factors, including overcrowding of patients, an insufficient number of healthcare professionals, high patient turnover, and communication failure among multi-professional teams. Therefore, the emergency service is a crucial site for the occurrence of problems [25]. Orthopedic patients typically receive pharmacological interventions to alleviate pain, with anti-inflammatory agents such as nonsteroidal anti-inflammatory drugs, opioid analgesics, and other central nervous system depressants, including gabapentin and pregabalin. Patients receiving care from the internal medicine service at our institution often present with complex diseases that require multiple medications. Thus, patients from these services were at increased risk for DDIs. Patients receiving radiotherapy experience a low prevalence of DDIs as the main medications were prescribed by other services. The prescriptions from the radiology department were mainly from the division of Nuclear Medicine where their prescriptions were in accordance with specific protocols and were generally not overly complicated, leading to the identification of this phenomenon as a protective factor.

The findings from this research support the development of clinical guidelines pertaining to clinically significant DDIs and their potential adverse outcomes, as well as management strategies for clinicians to facilitate the early recognition of potential DDIs. These strategies necessitate the use of multiple databases, a comprehensive literature review, and consultations with clinical pharmacists and expert clinicians. Their implementation can effectively prevent the omission of such interactions and encourage the administration of medication that is suitable for geriatric patients [4,14,19].

The strength of this study is the use of a reliable and well-established database from prior studies [4,11]. Additionally, it is the first study that investigates the prevalence of major DDI and its associated factors in a geriatric outpatient setting within a tertiary care hospital located in Thailand. It is important to acknowledge that there are some limitations to this study. Firstly, due to its retrospective nature, the data obtained from electronic medical records may be incomplete or underestimate certain aspects, such as the information regarding the real-life clinical complications of major DDI, diet, dosage form, duration of drug administration, and route of drug use. Moreover, data regarding the use of over-the-counter medications (OTC) or herbals was not collected. It is recommended to conduct further investigation using prospective study designs. Secondly, since this study was conducted in a single center, generalizability in different settings might be limited. Thirdly, it would be worthwhile to consider utilizing at least two databases to improve precision in detecting DDIs. Finally, the database has undergone frequent updates with regard to potential drug–drug interactions (DDIs). Thus, if certain drugs within each classification have been modified, the results of this study might deviate from those of previous versions.

## 5. Conclusions

Major drug–drug interaction was identified in about a third of patients among a geriatric outpatient setting of a tertiary care hospital. The risk of major DDI was found to increase in correlation with age, sex, polypharmacy, certain chronic diseases, and primary healthcare services. Anti-diabetic medications represented the class of major DDI that are commonly found. While the identification of significant drug–drug interactions using software may be too sensitive, it is essential to develop suitable strategies for improving the prescription process in geriatric patients to avoid missing any significant interactions.

## Figures and Tables

**Table 1:** Baseline data of studied patients.

Variables	No DDI (n = 3458, 31.8%)		Moderate DDI (n = 3414, 31.4%)		Major DDI (n = 4004, 38.8%)	
Age (years), mean (sd)	73.6	(6.4)	74.0	(7.2)	75.0	(6.7)
Gender, n(%)						
• Female	1519	(43.9)	1574	(46.2)	1876	(46.8)
• Male	1939	(56.1)	1840	(53.8)	2128	(53.2)
No.of medication (item),n(%)						
• 2–4	1868	(54.0)	1597	(46.8)	938	(23.4)
• 5–9	1512	(43.7)	1571	(46.0)	1923	(48.0)
• ≥10	78	(2.3)	246	(7.2)	1144	(28.6)
Principal diagnosis						
• Musculoskeletal disease	713	(20.6)	514	(15.1)	693	(17.3)
• Cancer	472	(13.7)	404	(11.8)	354	(8.8)
• Metabolic disease	464	(13.4)	741	(21.7)	904	(22.6)
• Cardiovascular disease	267	(7.7)	332	(9.7)	451	(11.3)
• CKD	240	(6.9)	161	(4.7)	342	(8.5)
• CNS disease	389	(11.3)	428	(12.5)	468	(11.7)
• Gastroduodenal disease	119	(3.4)	88	(2.6)	83	(2.1)
• Psychiatric disorders	55	(1.6)	101	(3.0)	114	(2.9)
• Airway disease	86	(2.5)	66	(1.9)	79	(1.9)
• Urosurgical disease	66	(1.9)	90	(2.6)	72	(1.8)
• Hepatobiliary disease	67	(1.9)	71	(2.1)	53	(1.3)
• TB	112	(3.3)	27	(0.8)	20	(0.5)
• Other diseases	408	(11.8)	391	(11.5)	372	(9.3)
No. of comorbid, mean(sd)	1.0	(0.8)	1.0	(0.8)	1.1	(0.9)
Primary medical service, n(%)						
• General practitioner	288	(8.3)	367	(10.7)	368	(9.2)
• Internal medicine	1878	(54.3)	1929	(56.5)	2473	(61.8)
• Orthopedics	599	(17.3)	440	(12.9)	531	(13.3)
• Surgery	230	(6.7)	178	(5.2)	169	(4.2)
• Psychiatry	87	(2.5)	158	(4.6)	171	(4.3)
• Emergency	118	(3.4)	109	(3.2)	153	(3.8)
• Palliative care	112	(3.2)	60	(1.8)	65	(1.6)
• ENT	67	(2.0)	44	(1.3)	34	(0.8)
• Radiology	37	(1.1)	91	(2.7)	13	(0.3)
• Gynecology	42	(1.2)	38	(1.1)	28	(0.7)

Note: DDI: drug–drug interaction; n: total numbers of patients; sd: standard deviation; OR: odds ratio; CI: confidence interval; No.: number; musculoskeletal disease included osteoarthritis, rheumatoid arthritis, and pain-in-joint stiffness; metabolic disease included DM, HT, and DLD; CNS disease included stroke, dementia, and epilepsy; CKD: chronic kidney disease; TB: tuberculosis; and ENT: ear nose throat.

**Table 2:** Common drugs associated with major DDI.

Interacting Pair (ATC Code)		Prevalence (%)	Summary [11]
Sulfonylureas	Dipeptidyl peptidase-IV inhibitors	3.95	Dipeptidyl peptidase-IV inhibitors may enhance the hypoglycemic effect of sulfonylureas.
Top 3 common drugs			
- Glipizide (A10BB07)	- Sitagliptin (A10BH01)	1.65	
- Glipizide (A10BB07)	- Linagliptin (A10BH05)	1.24	
- Glipizide (A10BB07)	- Gemigliptin (A10BH06)	0.50	
Opioid analgesics	CNS depressants	2.86	CNS depressants may enhance the CNS depressant effect of opioid analgesics.
Top 3 common drugs			
- Tramadol (N02AX02)	- Lorazepam (N05BA06)	1.01	
- Tramadol (N02AX02)	- Nortriptyline (N06AA10)	0.85	
- Morphine (N02AA01)	- Lorazepam (N05BA06)	0.32	
Salicylates	Nonsteroidal anti-inflammatory agents	2.77	Aspirin may enhance the adverse/toxic effect of nonsteroidal anti-inflammatory agents.
Top 3 common drugs			
- Salicylate (N02BA04)	- Etoricoxib (M01AH05)	0.96	
- Salicylate (N02BA04)	- Celecoxib (M01AH01)	0.94	
- Salicylate (N02BA04)	- Naproxen (M01AE02)	0.75	
Insulins	Dipeptidyl peptidase-IV inhibitors	2.51	Dipeptidyl peptidase-IV inhibitors may enhance the hypoglycemic effect of insulins.
Top 2 common drugs			
- insulin glargine (A10AE04)	Linagliptin (A10BH05)	0.93	
- insulin aspart (A10AB05)	Sitagliptin (A10BH01)	0.70	
HMG-CoA reductase inhibitors (statin)	Colchicine	2.40	Colchicine may enhance the myopathic (rhabdomyolysis) effect of HMG-CoA reductase inhibitors (statins).
Top 2 common drugs			
- Atorvastatin (C10AA05)	- Colchicine (M04AC01)	1.01	
- Simvastatin (C10AA01)	- Colchicine (M04AC01)	0.74	
Methotrexate	Nonsteroidal anti-inflammatory agents	1.82	Nonsteroidal anti-inflammatory agents may increase the serum concentration of methotrexate.
- Methotrexate (L04AX03)	- Naproxen (M01AE02)	1.17	
CYP2C19 Substrates (Strong)	CYP2C19 inducers (high risk with inducers)	1.16	CYP2C19 inducers (strong) may increase the metabolism of CYP2C19 substrates (high risk with inducers).
- Rifampicin (J04AB02)	- Omeprazole (A02BC01)	1.02	
Salicylates	Ginkgo biloba	0.86	Ginkgo biloba may enhance the anticoagulant effect of salicylates.
- Salicylate (N02BA04)	- Ginkgo biloba (N06DX02)		

Note: Major DDI: The effects of DDIs might be life-threatening or cause permanent damage; ATC: Anatomical Therapeutic Chemical (ATC) Classification.

**Table 3:** Factors associated with major DDI according to multiple logistic regression analysis.

Factors	Univariate			Multivariate		
	Crude OR	95%CI	*p*-Value	Adjusted OR	95%CI	*p*-Value
Age (years)	1.03	(1.02–1.04)	<0.05	1.03	(1.02–1.03)	<0.05
Female	1.08	(0.99–1.16)	0.07	1.13	(1.03–1.23)	<0.05
No.of medication (item)						
• 2–4	1	-	-	1	-	-
• 5–9	2.30	(2.10–2.53)	<0.05	2.27	(2.06–2.49)	<0.05
• ≥10	13.04	(11.31–15.05)	<0.05	13.78	(11.87–15.99)	<0.05
Principal diagnosis						
• Musculoskeletal disease	1.21	(1.04–1.42)	<0.05	1.37	(1.13–1.65)	<0.05
• Cancer	0.87	(0.73,1.03)	0.11	1.08	(0.88–1.32)	0.46
• Metabolic disease	1.61	(1.39–1.87)	<0.05	1.89	(1.59–2.24)	<0.05
• Cardiovascular disease	1.62	(1.36–1.92)	<0.05	1.51	(1.24–1.84)	<0.05
• CKD	1.83	(1.52–2.21)	<0.05	1.08	(0.87–1.35)	0.48
• CNS disease	1.23	(1.04–1.45)	<0.05	1.24	(1.02–1.49)	<0.05
• Gastroduodenal disease	0.86	(0.65–1.14)	0.30	0.93	(0.68–1.28)	0.66
• Psychiatric disorder	1.57	(1.20–2.06)	<0.05	1.79	(1.19–2.57)	<0.05
• Airway disease	1.12	(0.83–1.50)	0.47	1.20	(0.86–1.66)	0.28
• Urogenital disease	0.99	(0.73–1.35)	0.96	0.77	(0.54–1.10)	0.16
• Hepatobiliary disease	0.82	(0.59–1.16)	0.27	1.02	(0.70–1.48)	0.91
• TB	0.31	(0.19–0.50)	<0.05	0.18	(0.11–0.33)	<0.05
• Other diseases	1	-	-		-	
No. of comorbid	1.10	(1.05–1.15)	<0.05	0.98	(0.93–1.04)	0.51
Main medical service						
• General practitioner	1		-		-	
• Internal medicine	1.16	(1.01–1.33)	<0.05	1.29	(01.09–1.51)	<0.05
• Orthopedics	0.91	(0.77–1.07)	0.26	1.36	(1.06–1.65)	<0.05
• Surgery	0.74	(0.59–0.92)	<0.05	0.99	(0.77–1.29)	0.95
• Psychiatry	1.24	(0.98–1.57)	0.07	1.25	(0.89–1.75)	0.20
• Emergency	1.20	(0.94–1.53)	0.14	1.72	(1.20–2.11)	<0.05
• Palliative care	0.67	(0.49–0.92)	<0.05	1.10	(0.76–1.85)	0.62
• ENT	0.55	(0.36–0.82)	<0.05	0.85	(0.54–1.34)	0.48
• Radiology	0.18	(0.10–0.32)	<0.05	0.45	(0.23–0.79)	<0.05
• Gynecology	0.62	(0.40–0.98)	<0.05	0.80	(0.49–1.32)	0.39

Note: OR: odds ratio; CI: confidence interval; No.: number; metabolic disease included DM, HT, and DLD; CNS disease included stroke, dementia, and epilepsy; CKD: chronic kidney disease; TB: tuberculosis; and ENT: ear nose throat.

## Data Availability

Data are contained within the article.
